# Facilitating Understanding, Modeling and Simulation of Infectious Disease Epidemics in the Age of COVID-19

**DOI:** 10.3389/fpubh.2021.593417

**Published:** 2021-02-12

**Authors:** David M. Rubin, Shamin Achari, Craig S. Carlson, Robyn F. R. Letts, Adam Pantanowitz, Michiel Postema, Xriz L. Richards, Brian Wigdorowitz

**Affiliations:** ^1^Biomedical Engineering Research Group, School of EIE, University of the Witwatersrand, Johannesburg, South Africa; ^2^BioMediTech, Faculty of Medicine and Health Technology, Tampere University, Tampere, Finland

**Keywords:** system dynamics, epidemic modeling, undergraduate teaching, engagement with COVID-19 models, mechanistic epidemiology

## Abstract

Interest in the mathematical modeling of infectious diseases has increased due to the COVID-19 pandemic. However, many medical students do not have the required background in coding or mathematics to engage optimally in this approach. System dynamics is a methodology for implementing mathematical models as easy-to-understand stock-flow diagrams. Remarkably, creating stock-flow diagrams is the same process as creating the equivalent differential equations. Yet, its visual nature makes the process simple and intuitive. We demonstrate the simplicity of system dynamics by applying it to epidemic models including a model of COVID-19 mutation. We then discuss the ease with which far more complex models can be produced by implementing a model comprising eight differential equations of a Chikungunya epidemic from the literature. Finally, we discuss the learning environment in which the teaching of the epidemic modeling occurs. We advocate the widespread use of system dynamics to empower those who are engaged in infectious disease epidemiology, regardless of their mathematical background.

## Introduction

Compartmental modeling of infectious disease epidemic behavior in terms of differential equations depends on so-called dynamical or mechanistic epidemiology, as distinct from classical epidemiology (1, 2). Both approaches have great utility. However, the skills needed to engage with the dynamical approach, include coding and fluency in the mathematics of differential equations. These are frequently absent in undergraduate and postgraduate courses (1). This is compounded by the fact that models of epidemics tend to be non-linear (2).

As the COVID-19 pandemic sweeps the world, it seems likely that an interest in mechanistic modeling will grow. Indeed, compartmental modeling of the spread of the COVID-19 pandemic (3–9), has become critical in our efforts to address the crisis. However, undergraduate medical students are not typically equipped with the tools to follow the details of such research let alone to perform their own modeling.

Bellan et al. (2) addressed this by gamifying epidemiology teaching in a manner reminiscent of the beer distribution game (10). Handel addressed this by developing a set of epidemic simulators for the R programming language (1). While both gamification and so-called epidemic flight simulators are valuable pedagogical tools, we suggest extending this to facilitate independent modeling by students who lack experience in differential equations.

The question we address in this discussion is: can students of epidemiology who have little or no background in differential equations or coding, engage meaningfully with the process of constructing models? Remarkably, the answer to this is a resounding yes. To achieve this, we propose the adoption of system dynamics in epidemiology education.

Our advocacy for a system dynamics approach to address the teaching of deterministic models of epidemics is only one of many modeling paradigms and approaches. For example, stochastic models, including agent-based models (ABM) offer an alternative approach, and they hold advantages in modeling the early phase of an epidemic while there are still small numbers of infected individuals, and also when the demographic and environmental parameters are subject to variability over the course of the epidemic (11).

Allen (11) showed the utility of these models for Susceptible, Infected, Recovered (SIR) behavior and for transmission of malaria involving both humans and mosquitoes. He compared them to deterministic models. However, these models tend to require mathematical sophistication and a deep understanding of the underlying probability distributions which would make them less suitable for students who do not have a formal mathematical background.

ABM have been found to be very useful to model respiratory epidemics (12). With the availability of user-friendly ABM software, the possibility of including this approach in an introductory course may be considered.

## Pedagogical Principles

Forrester developed the discipline of system dynamics, wherein all models are expressed in terms of quantities known as stocks (levels) and flows (rates) (13). The creation of stock-flow diagrams is analytically identical to writing the equivalent set of coupled first-order differential equations, and facilitates the modeling of complex, non-linear systems in a highly intuitive and accessible manner.

Galea advocates the adaptation of old methods and the adoption of new ones in epidemiology (14). System dynamics falls into the category of long-established methods with as yet untapped potential in the educational sphere of epidemiology and public health. Indeed, Galea et al. point out the importance of mathematical models dealing with complexity, feedback and causality (15), all of which may be addressed by system dynamics approaches.

Consider the simple example of water accumulating in a bathtub. The accumulated quantity, known as the stock, is the water in the tub. This stock results from the inflows and outflows representing the rates of change of the stock known as flows. Other examples of stocks include the inventory of vaccines in a rural clinic, and the number of infected individuals during an epidemic.

In the vaccine example, the flows would be the rates of vaccine delivery and usage, respectively. In the case of infected individuals, the flows represent the rates of infections and recovery. In each case, the stock is the accumulation of the difference between the inflow and outflow rates.

**Figure 1:**
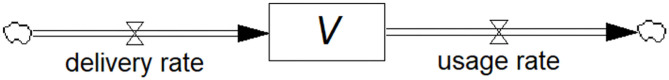
Stock-flow diagram showing the model of a vaccine (stock) being replenished through the deliveries and depleted through usage (flows). This is a visual representation of the associated differential equation.

Figure 1 shows the stock-flow diagram of the flows called delivery rate (*R*_delivery_) and usage rate (*R*_usage_) of vaccine represented by pipe-like arrows with a valve symbol indicating variable flow. The diagrams in this paper are constructed in the Personal Learning Edition (PLE) version of the system dynamics software Vensim® (Ventana Systems, Inc., Harvard, MA, USA) which is available free of charge for educational use. The accumulating or diminishing stock of vaccines is represented by the rectangular box. Anyone with an interest in the field can construct these diagrams, yet they may be surprised to know that they have created a differential equation, i.e., the rate of change of the stock of vaccines, d*V*/d*t*, is equal to the difference between the inflow and outflow rates. Mathematically:

(1) $$\begin{array}{l} \frac{\text{d}V}{\text{d}t}=R_{\text{delivery}}-R_{\text{usage}}. \end{array}$$

The two flows and the stock can be populated with numerical values and units, after which the simulation can be run. This produces graphical output allowing us to examine the behavior of vaccine stock.

### System Dynamics Applied to Compartmental Models of Infectious Epidemics

Many authors, for example Ford (13), have used system dynamics to implement an SIR model. We provide a brief description of this model's implementation in Vensim PLE.

**Figure 2:**
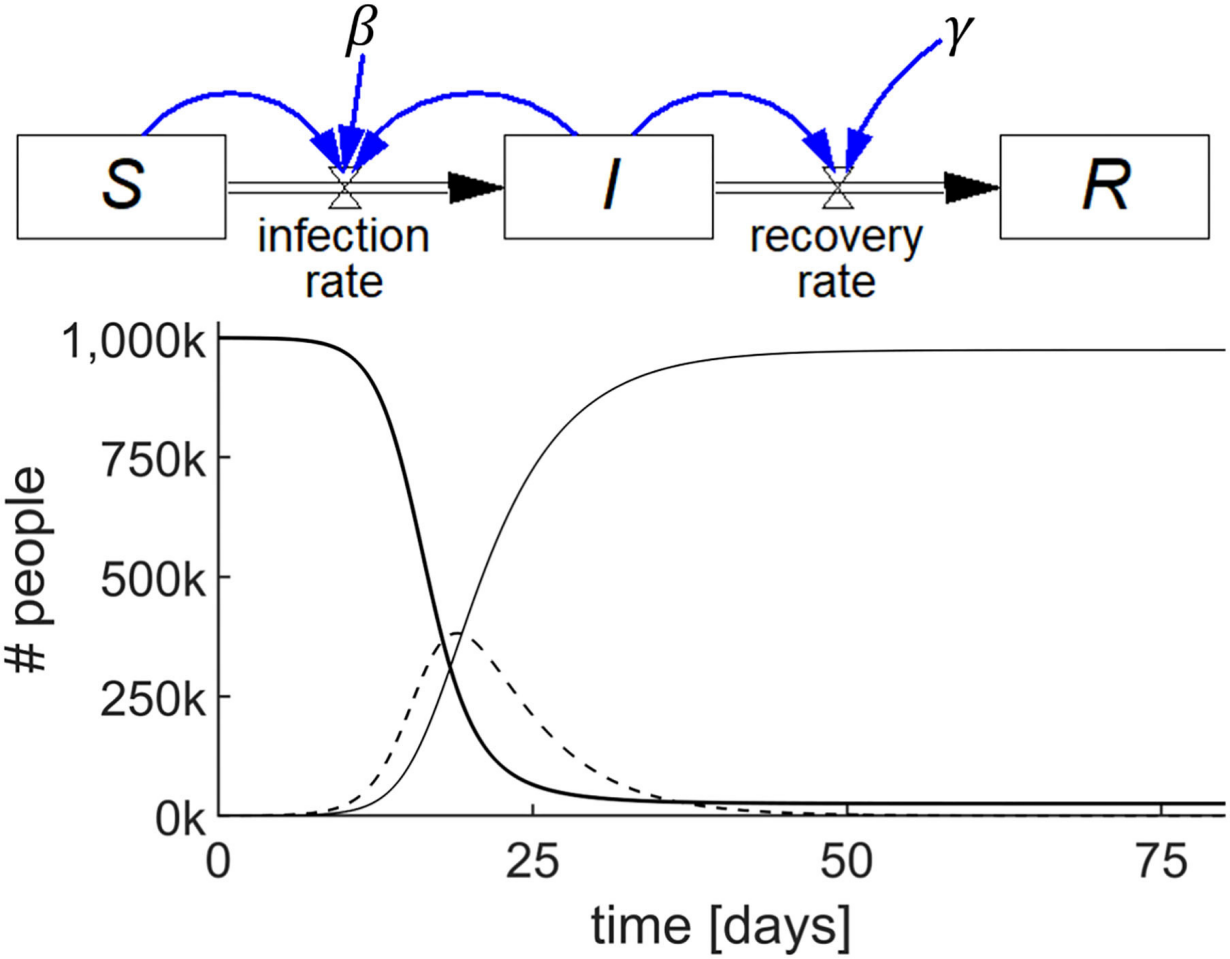
Stock-flow diagram of the SIR epidemic model representing the three differential equations (top) and results of the simulation (bottom) showing the behavior of *S* (thick line), *I* (dashed line), and *R* (thin line) with parameters *S*(0) = 10^6^; *I*(0) = 100; *R*(0) = 0; β = 0.75/day; and γ = 0.2/day.

This SIR model comprises three accumulations, *viz*. *S, I*, and *R*, each represented by a stock as shown in Figure 2 (top). For this example, we will assume zero births and deaths.

We begin by examining how the stock *S* changes. *S* has zero inflow rate and becomes depleted as people become infected and convert to *I*. Let us call the flow out of *S*, and into *I*, the *infection rate*, which is known to be equal to the product of a constant (β), the proportion of infected individuals (*I/N*), where *N* is total population size, and *S*. Stated succinctly, *infection rate* = β*(I/N)S*.

As the inflow to *S* is zero and thus *infection rate* is the only flow affecting *S*, we conclude that the rate of change of *S*, d*S*/d*t*, is 0 – β*(I/N)S*. A mathematician would state this as the first differential equation of the SIR model as follows:

(2) $$\begin{array}{l} \frac{\text{d}S}{\text{d}t}=-\beta \left(\frac{I}{N}\right)S. \end{array}$$

The stock, *S*, and its outflow, *infection rate*, shown in Figure 2 (top), is the highly visual system dynamics way of stating this first differential equation of the SIR model.

For the infected stock *I*, the rate at which *I* changes is the difference between the inflow to *I*, which is the *infection rate* = β*(I/N)S*, and the outflow from *I*, which we will call the *recovery rate*. The *recovery rate* is equal to the product of a constant, γ, and *I*, i.e., *recovery rate* = γ*I*. Stated in words, the rate of change of *I* is equal to the *infection rate* minus the *recovery rate*, which is also shown in Figure 2 (top) by the stock, *I*, and its inflow and outflow. Stated mathematically:

(3) $$\begin{array}{l} \frac{\text{d}I}{\text{d}t}=\beta \left(\frac{I}{N}\right)S-\gamma I. \end{array}$$

Finally, we examine what brings about the rate of change of the stock of recovered people, *R*. As there is no outflow from *R* in this model, the rate of change of *R* must simply be the *recovery rate* = γ*I*, which is again shown visually in Figure 2 (top). Mathematically, this is:

(4) $$\begin{array}{l} \frac{\text{d}R}{\text{d}t}=\gamma I. \end{array}$$

Thus, Figure 2 (top) represents the SIR model in a way which circumvents the need to write the differential equations. Each of the three stocks, *S, I*, and *R*, with their accompanying in and out flows, represent the respective differential equations. Once populated with numerical values, including the initial values for the stocks, the model may be simulated. The output is shown graphically as in Figure 2 (bottom). Details of this model's construction and simulation can be found in the video SIR_epidemic.mp4 in the Supplementary Material.

**Figure 3:**
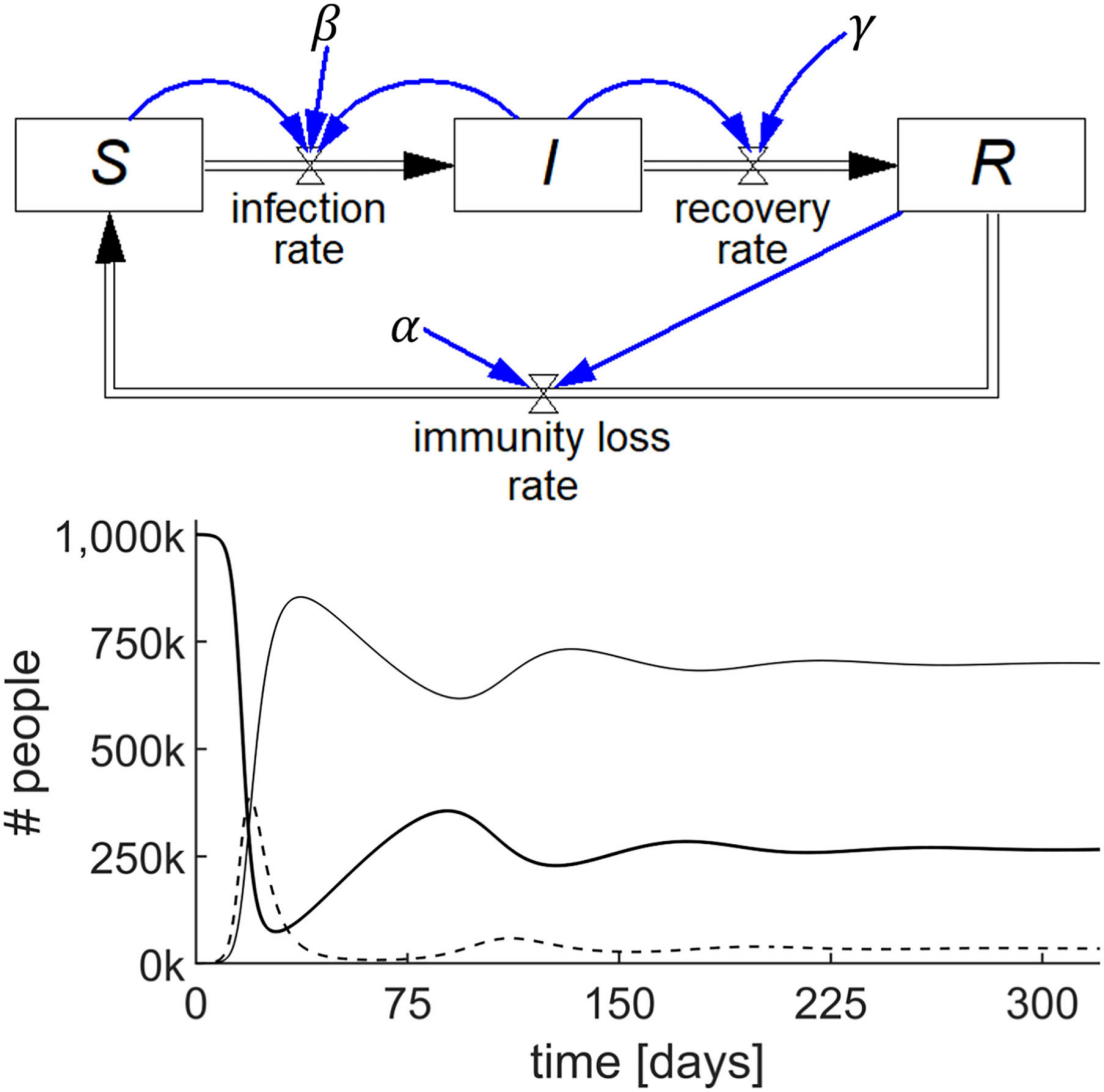
Stock-flow diagram of the SIRS epidemic model representing the three differential equations (top) and results of the simulation (bottom) showing the behavior of *S* (thick line), *I* (dashed line), and *R* (thin line) with parameters *S*(0) = 10^6^; *I*(0) = 100; *R*(0) = 0; β = 0.75/day; γ = 0.2/day; and α = 0.01/day.

A SIRS model is shown in Figure 3 (top), where the recovered people gradually lose immunity at a rate that is proportional to *R* with a constant α, i.e., *immunity loss rate* = α*R*. This waning immunity requires a modification of two of the stocks because α*R* becomes an inflow to *S* and an outflow from *R*. The output of this simulation for a specific set of numerical parameters and initial values is shown in Figure 3 (bottom).

**Figure 4:**
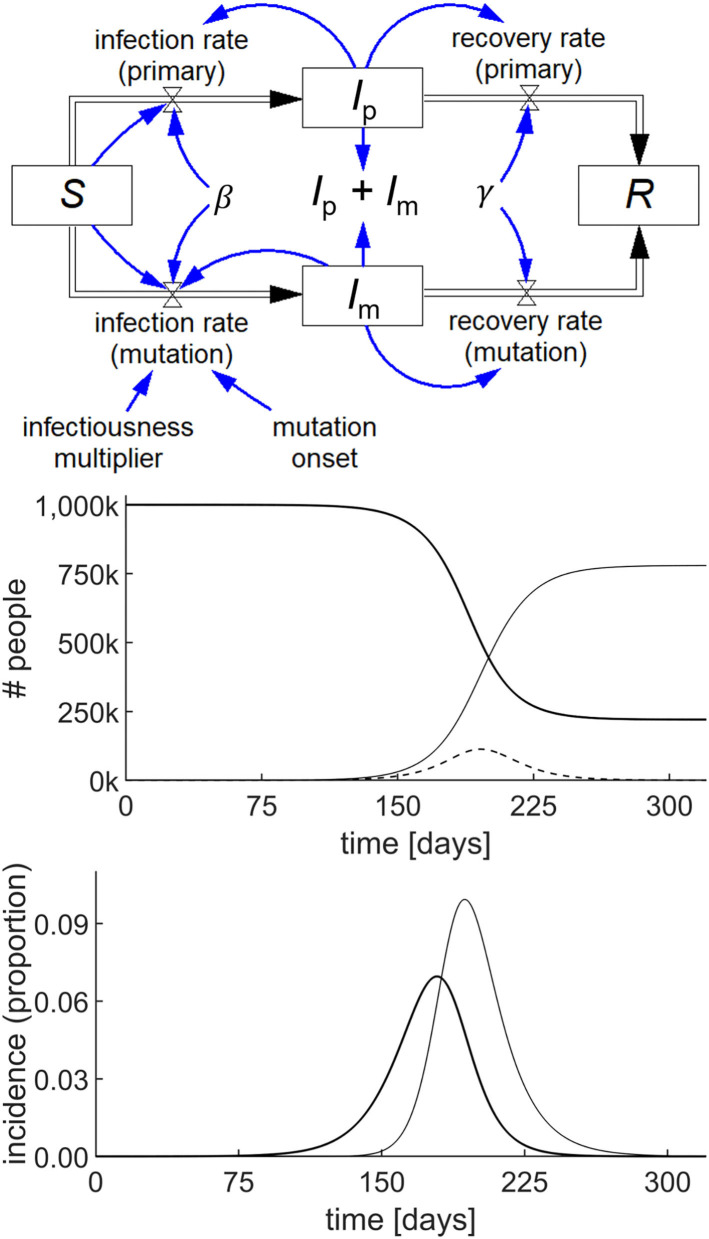
Stock-flow diagram of a simple COVID-19 model with a mutation starting at 60 days, based on the SIR epidemic model (top) and the result of the simulation (middle) showing the behavior of *S* (thick line), *I*_p_ + *I*_m_ (dashed line), and *R* (thin line) with parameters *S*(0) = 10^6^; *I*_p_(0) = *I*_m_(0) = 1; *R*(0) = 0; β = 0.19/day; γ = 0.125/day; and infectiousness multiplier = 1.5. The incidence graph (bottom) shows the primary infection rate (thick line) and mutation infection rate (thin line) per 100,000 people.

The utility of System Dynamics in COVID-19 is demonstrated by modeling the behavior of the epidemic when a single instance of a mutated variant arises *de novo* on day 60 of the simulation. The mutant strain is assumed to be 50% more infectious than the primary strain. A standard pair of SIR models with common *S* and *R* are used, one for the primary virus in the infected population, *I*_p_ and the other for the mutant variant in the infected population, *I*_m_. With the specific parameters chosen as shown in Figure 4, the mutant strain becomes the dominant variant.

A number of fundamental epidemiological concepts can be elucidated through system dynamics models. For example, our students are typically introduced to the concepts of incidence rate and point prevalence at various times during their study of public health. However, these concepts can be understood from the graphical output of *infection rate* and the stock *I*, respectively. These can be scaled to *S* or *N*, respectively. Another example of conceptual understanding is the basic reproduction number, *R*_0._ This can be explored by experimenting with the numerical values in the model to determine under what conditions the epidemic fails to ignite. This gives students an intuitive sense of the behavior of epidemics with various values for *R*_0_. and the parameters that influence *R*_0_. While fundamental to the study of the spread of epidemics in populations, the basic reproduction number has also found application in a complex mathematical model describing the dynamics of the HTLV-1 virus in the body in the face of a cellular immune response (16).

Highly nuanced epidemic models have been proposed, for example the application of an SIR model to a dynamic network topology in the presence of inter-city commuting and varying populations (17). Other models include the influence of the spread of awareness about an epidemic with a resulting interaction between the two layers of awareness and infection (18). This idea has been extended to take account of the influence of the spread of various types of behavior-modifying information, both positive and negative, over social networks in the setting of real-world topologies (19). While a variety of modeling approaches will generally be required to address this level of complexity, it is certainly possible to build limited deterministic models of these systems using system dynamics in order to appreciate their essential characteristics.

One of the objectives in teaching system dynamics to medical students is to further an understanding of published articles and to facilitate access to a domain, which would otherwise be too mathematically abstract for their training. To appreciate the utility of the system dynamics approach in this objective, it is useful to examine an epidemic dynamical model in the literature and compare it to the equivalent system dynamics implementation.

Renault et al. (20) reported on field data from the 2005/2006 Chikungunya epidemic on Réunion Island, and Yakob and Clements (21) developed a deterministic model of this epidemic comprising eight coupled ordinary differential equations, which includes both the human host and mosquito vector populations. In addition to *S, I*, and *R*, they include two additional human host stocks, *viz*. exposed (*E*) and asymptomatically infected (*I*_a_), and three mosquito stocks, *viz*. susceptible (*X*), exposed (*Y*), and infected (*Z*). All other symbols represent constants.

The eight differential equations of the Yakob–Clements model are (21):

(5) $$\begin{array}{l} \frac{\text{d}S}{\text{d}t}=-\beta_{1}SZ; \end{array}$$

(6) $$\begin{array}{l} \frac{\text{d}E}{\text{d}t}=\beta_{1}SZ-\lambda_{1}E; \end{array}$$

(7) $$\begin{array}{l} \frac{\text{d}I}{\text{d}t}=\phi \lambda_{1}E-\gamma I; \end{array}$$

(8) $$\begin{array}{l} \frac{{\text{d}I}_{a}}{\text{d}t}=\left(1-\phi \right)\lambda_{1}E-\gamma I_{\text{a}}; \end{array}$$

(9) $$\begin{array}{l} \frac{\text{d}R}{\text{d}t}=\gamma \left(I+I_{\text{a}}\right); \end{array}$$

(10) $$\begin{array}{l} \frac{\text{d}X}{\text{d}t}=\mu -\beta_{2}X\left(I+I_{\text{a}}\right)-\mu X; \end{array}$$

(11) $$\begin{array}{l} \frac{\text{d}Y}{\text{d}t}=\beta_{2}X\left(I+I_{\text{a}}\right)-\lambda_{2}Y-\mu Y; \end{array}$$

(12) $$\begin{array}{l} \frac{\text{d}Z}{\text{d}t}=\lambda_{2}Y-\mu Z. \end{array}$$

**Figure 5:**
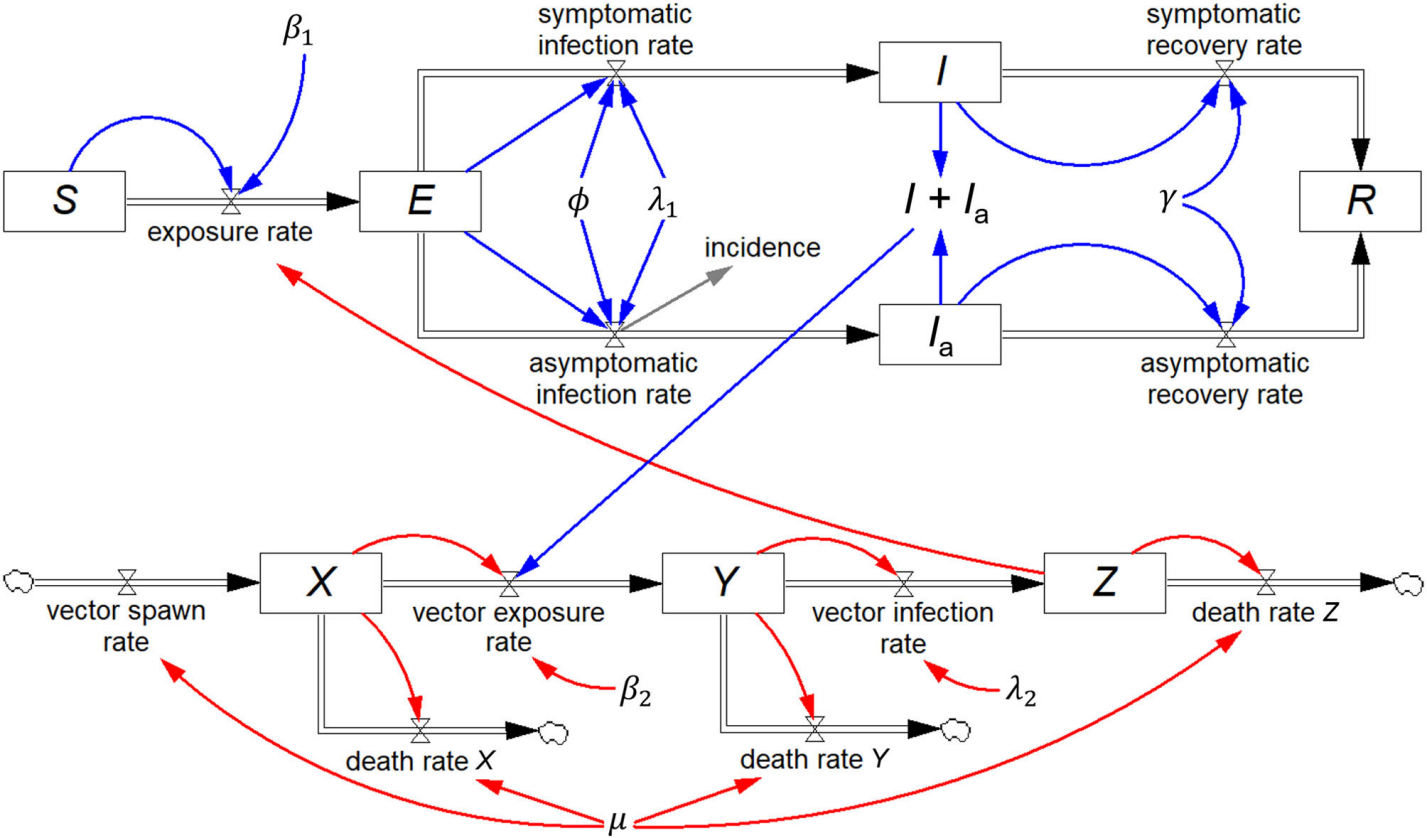
Stock-flow diagram of the Yakob–Clements model (21) of the 2005/2006 La Réunion Island Chikungunya epidemic. This is a visual representation of the eight associated coupled first-order differential equations.

The equivalent system dynamics model in Figure 5 represents an alternative to working directly with differential equations to recreate the Yakob–Clements model. The eight stocks, each with their respective inflows and outflows constitute a visual representation of the eight differential equations.

**Figure 6:**
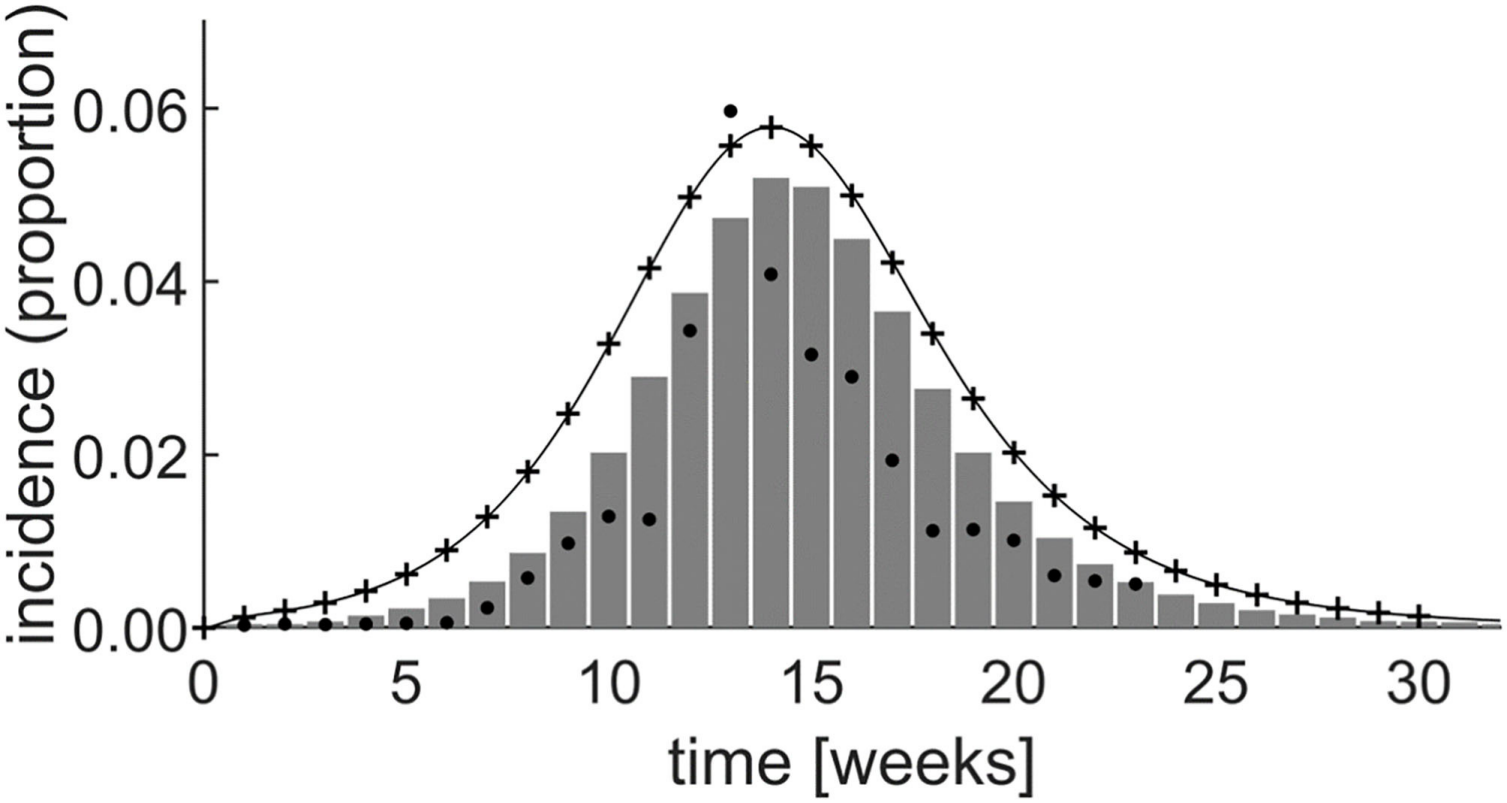
Simulation results of the Yakob–Clements model (21) using system dynamics (thin line). The MATLAB® simulation results (plusses) confirm the system dynamics results, demonstrating the equivalence between system dynamics and direct differential equation modeling. The Yakob–Clements model (21) simulation results (bars) and the field data (20, 21) (dots) are superimposed for comparison. Week 1 corresponds to week 45 of 2005 in Yakob and Clements' paper (21).

Figure 6 shows the incidence rate generated by both the system dynamics model and by simulating the equivalent set of differential equations in Matlab (The Mathworks, Nattick, MA, USA). This is superimposed on a graph of the incidence rate produced by Yakob and Clements (21) and field data from the epidemic (20, 21). By experimentally adjusting the model parameters, initial stock values, and the level of prior herd immunity, the system dynamics simulation achieved a good fit with the field data, as shown in Figure 6.

The expected exact equivalence between the system dynamics model output and the output produced by directly simulating the differential equations in Matlab when using the same parameters and initial values, is evident (Figure 6). Moreover, as reproduced in Figure 6, the Yakob–Clements model (21) produced a good fit with the field data collected during the Chikungunya epidemic on Réunion Island (20).

As seen in Figure 6, we too were able to achieve a good fit to the data by implementing the Yakob–Clements model in Vensim PLE and Matlab. However, the PLE version of Vensim, which is the primary teaching tool discussed in this paper, does not support model calibration, and the fit was achieved by eye. This may, in part, explain the discrepancy between the output of the original Yakob–Clements model (21) and our implementations. We point out that model calibration is a higher-order process which is not included in our introductory course for medical students. Also, in our implementations, we determined incidence directly from the conversion rate of the exposed compartment (*E*) to symptomatic infected compartment (*I*) at each incremental time step. Yakob and Clements determined the incidence by summing the daily incidence every 7 days, which facilitated fitting their model to the weekly incidence data acquired in the field study (20).

## Learning Environment

The application of system dynamics to epidemic modeling is taught within a larger context in a course entitled Health System Dynamics (HSD). HSD is run for undergraduate first-year students in both the 6-year medical school curriculum and the 3-year medical science curriculum. Most of our students have not studied mathematics beyond high school level. Thus, other than the few students who have taken advanced program mathematics in high school or other advanced instruction in mathematics, most are acquainted with differential calculus but not with integral calculus or differential equations. This is compounded by the fact that our health sciences curricula do not include any further courses in mathematics.

The class sizes for both groups are nominally 200 students per group, and the teaching of such large classes is challenging. We have produced an online edX course called System Dynamics for Health Sciences, and we use this course as part of a flipped classroom model. Students watch the videos and attempt the exercises. The students subsequently attend tutorial sessions conducted by senior academic staff, where they are able to raise any aspects of the course and their questions are discussed in a group session. In this way, most of the group benefits from the questions and the direction of the ensuing discussion is guided largely by the students' questions. This combined lecture/tutorial style has the advantage of being student-driven.

The laboratory aspect of the course requires approximately 3 h/week of student time. The students use Vensim PLE on workstations to solve tasks requiring the development and analysis of models, including epidemic examples. These laboratory sessions are usually conducted by junior teaching assistants who provide assistance with both the modeling process and with any difficulties students may have with use of the software.

Assessment involves theory and a laboratory task and students have frequently been required to perform epidemic modeling in the laboratory aspect of the examination.

## Discussion and Conclusion

Dynamical (mechanistic) methods in infectious disease epidemiology are important tools in the epidemiologist's armamentarium, however, many students don't have an adequate background in coding and differential equations to engage in dynamical modeling.

System dynamics offers a highly intuitive approach to modeling in epidemiology. It provides a visual representation of dynamic phenomena in epidemiology in terms of two basic elements, *viz*. the rates of conversion from one population category to another called flows (rates), and the accumulated populations in each category called stocks (levels).

Building models of epidemics in system dynamics is intuitive and easy, yet the models actually represent sets of coupled first-order differential equations. However, the system dynamics methodology is readily accessible to students of epidemiology who do not have a background in differential equations or coding.

Unlike the use of so-called flight simulators, which facilitate simulation of epidemics with user-chosen numerical constants, system dynamics allows the student of infectious disease epidemiology to engage with the process of creating their own models. This intuitive approach facilitates model development in a manner, which is equivalent to working directly with the differential equations.

An ability to engage with deterministic models affords undergraduate medical students a far greater participation in the public health debates on epidemics, which are frequently dependent on mathematical models. The COVID-19 pandemic has highlighted this need for greater modeling capacity among medical professionals. In addition to mathematically naïve students, the system dynamics approach may also have utility for the community of experienced modeling practitioners by facilitating a rapid prototyping environment for the development and exploration of complex models. The ability to create a model in a way that explicitly shows the accumulations and flows has great advantages both in terms of iteratively exploring model designs, and also in terms of demonstrating the models to policymakers and other interested stakeholders.

## Data Availability Statement

The datasets for the Chikungunya epidemic are publicly available from references (20, 21). No other input data were used in this article. The model-generated output datasets are available on request from David M. Rubin, at david.rubin@wits.ac.za.

## Author Contributions

DR initiated the course, proposed this paper and wrote the first draft. DR, SA, CC, RL, AP, XR, and BW were involved in developing and presenting and/or assessing the course and contributed to the technical content and development of the manuscript. MP reviewed the differential equations and contributed to the implementation of the multi-compartmental modeling example as well as contributing to the development of the manuscript. All authors contributed to the article and approved the submitted version.

## Conflict of Interest

The authors declare that the research was conducted in the absence of any commercial or financial relationships that could be construed as a potential conflict of interest.
